# Urinary retention in female OAB after intravesical Botox injection: who is really at risk?

**DOI:** 10.1007/s00192-016-3212-4

**Published:** 2016-11-26

**Authors:** Pawel Miotla, Rufus Cartwright, Katarzyna Skorupska, Michal Bogusiewicz, Ewa Markut-Miotla, Konrad Futyma, Tomasz Rechberger

**Affiliations:** 1 2nd Department of Gynaecology, Medical University of Lublin, ul. Jaczewskiego 8, 20-954 Lublin, Poland; 2 Department of Epidemiology and Biostatistics, Department of Urogynaecology, Imperial College London, London, UK; 3 Department of Paediatric Pulmonology and Rheumatology, Medical University of Lublin, Lublin, Poland

**Keywords:** Botulinum toxin A, Botox, Overactive bladder, Residual urine volume, Urgency incontinence, Urine retention

## Abstract

**Introduction and hypothesis:**

Intravesical onabotulinumtoxinA (Botox) injections are effective for the treatment of idiopathic overactive bladder (OAB) symptoms. The aim of our study was to assess the predisposing factors for urinary retention in women with OAB after intravesical Botox injection.

**Methods:**

All participants were women of European descent with idiopathic OAB. OnabotulinumtoxinA (100 U) was administered in 20 intra-detrusor injections. Analysis was performed based on the results of safety assessments made during follow-up (FU) visits on weeks 2, 4 and 12, in 208 women who were treated with Botox injections for refractory OAB and who completed all FU visits.

**Results:**

Women who required clean intermittent self-catheterisation (CISC) and those with post-void residual (PVR) greater than 200 ml were older in comparison with patients with PVR between 50 and 200 ml. Patients who required CISC were also characterised by higher parity and particularly by a higher number of vaginal deliveries. Other factors such as body mass index or comorbidities did not significantly influence PVR and the risk of CISC.

**Conclusions:**

Elderly and/or multiparous women are at increased risk of urinary retention after intravesical 100-U Botox injections. The risk of new onset urine retention in our study has completely disappeared 2 weeks after Botox injections. Based on our results of the way in which the PVRs have changed over time, we can conclude that OAB patients should be optimally assessed during the first 2 weeks after Botox injections.

## Introduction

An International Urogynecological Association (IUGA) and International Continence Society (ICS) joint report on the terminology for female pelvic floor dysfunction have defined overactive bladder (OAB) syndrome as urinary urgency, usually accompanied by frequency and nocturia, with or without urgency urinary incontinence, in the absence of urinary tract infection or other obvious pathological condition [1]. The prevalence of OAB in the population is over 16 % and it increases with age [2]. Moreover, symptoms of OAB affect more than 30 % of elderly patients [3]. Following behavioural therapy anticholinergic agents and/or mirabegron are then the mainstay of pharmacological treatment of OAB. However, in non-responders who are inadequately managed with such oral therapies there is a need for the intravesical injections of onabotulinumtoxinA (Botox) [4]. The National Institute for Health and Care Excellence (NICE) guidelines highlight that before invasive treatments, which include intravesical botulinum toxin injections, women with refractory OAB should undergo a multidisciplinary team review to ensure that all other non-invasive treatment options have been exhausted [5]. Adverse effects including the potential risk of urinary retention requiring catheterisation are among the greatest concerns for OAB patients considering Botox injections [6].

The efficacy of Botox (100 U; Allergan, Dublin, Ireland) in the treatment of idiopathic OAB has been proven in several randomised clinical trials; however, the risk factors for the occurrence of urinary retention after Botox injections are still not well recognised [7–10]. One multicentre, placebo-controlled, dose-ranging study (doses of 50 to 300 U) reported a dose-dependent proportion of patients with a post-treatment post-void residual (PVR) of 200 ml or greater. In patients who were treated at a dose of 100 U the percentage of clean intermittent self-catheterisation (CISC) was 10.9 %, whereas in the group treated with 200 U, the rate was almost double (21.2 %) [10].

The results of a long-term extension study, which included female and male patients, has found that the rate of de novo CISC was 4.0 % after the first Botox (100 U) injection cycle. Interestingly, patients who do not need to self-catheterise after their first Botox treatment are at a lower risk of needing CISC in later treatment cycles. The rate of CISC due to urinary retention was <2.0 % in all subsequent re-injection cycles [7]. One previous study has confirmed the association between increased preoperative PVR with urine retention after Botox injections [11].

The results of randomised clinical trials (RCTs) and non-randomised observational studies do not describe the factors for the prediction of urine retention after Botox (100U) injections in patients with refractory OAB very well [8–11]. The possibility of forecasting the risk of urinary retention after Botox injections could facilitate shared decision-making for third-line treatment options for refractory OAB. The aim of our observational study was to assess the predisposing factors for urinary retention in OAB women after intravesical Botox (100 U) injections.

## Materials and methods

The study protocol was approved by the local ethics committee. All participants were women of European descent. From February 2009 to November 2015, a total of 252 patients with refractory OAB were included in this prospective study. Analysis was performed based on the results of safety assessments made during follow-up (FU) visits on weeks 2, 4, 12 and at any other time in patients with clinically relevant urinary retention depending on need, in 208 women who were treated with Botox injections and who completed all FU visits. Patients were informed about potential adverse events of Botox and written consent was obtained from all study participants. Inclusion and exclusion criteria are summarised in Table 1. Patients were qualified for the treatment based on the results of 3-day bladder diaries.

**Table 1:** Inclusion and exclusion criteria of the study

Inclusion criteria	Exclusion criteria
Non-pregnant women over 18 years of age	Previous onabotulinumtoxinA injections to treat urological conditions
Idiopathic overactive bladder wet symptoms:	Contraindications to onabotulinumtoxinA use
≥8 micturitions/24 h	Contraindications to clean intermittent self-catheterisation
≥1 urgency urinary incontinence/24 h	Allergy to lidocaine
Lack of efficacy (at least two drugs, each for ≥1 month) or intolerance of antimuscarinic therapy and/or lack of efficacy or intolerance of mirabegron (≥1 month)	Previous anti-incontinence or prolapse surgery
Stage 0 or 1 on pelvic organ prolapse quantification (POP-Q) scale	Stress or mixed urinary incontinence
Maximum flow (Q-max) on uroflowmetry > 15 ml/s	Painful bladder syndrome
	Atrophic vaginitis
	Urinary tract infection
	Bladder or pelvic tumours and/or stones
	Neurological disorders affecting bladder function
	Post-void residual >100 ml before treatment
	Uncontrolled systemic disease, i.e. diabetes
	Stage > 2 on the POP-Q scale

Two percent lidocaine solution was infused into the bladder for at least 20 min before each procedure. Botox was administered by one of three investigators, dissolved into 10 ml of 0.9 % saline chloride and given in trigone-sparing injections in 20 sites during rigid cystoscopy under local anaesthesia. PVR measurements were checked with a single, three-dimensional, abdominal ultrasound in all patients before each procedure and at each follow-up visit. Urine dipstick testing was performed to screen for urinary tract infection (UTI). For patients with positive urinalysis, urine cultures were performed, and treatment of presumptive UTI was implemented. The patients were separated into five subgroups based on the results of PVR (residual urine volume: < 50 ml; 51–100 ml; 101–200 ml; 201–350 ml; urine retention requiring CISC) assessed at week 2 of follow-up. The incidence and severity of urine retention after Botox injections were assessed by two blinded evaluators. All patients with PVR greater than 350 ml were advised to start CISC. Patients with PVR <350 ml with significant symptoms of incomplete bladder emptying or voiding difficulties were also advised to perform CISC. Patients who performed CISC were checked using abdominal ultrasound in the out-patient clinic every 7–10 days. Moreover, the kidney scans were performed in all patients with urine retention because of the risk of upper urinary tract dilation and renal insufficiency. Patients on CISC took oral prophylaxis (furagin or ciprofloxacin) to prevent urinary tract infection. CISC was stopped when the patient had no clinical symptoms and PVR as measured by ultrasound was less than 300 ml.

The primary outcome measures included urinary retention, defined as the necessity for CISC, and PVR volumes after Botox injections. All associated factors (age, BMI, parity, comorbidities), which could potentially influence primary outcomes, were assessed using multivariate logistic regression tests. The secondary outcomes measure includes the duration of CISC and its association with potential risk factors for CISC. Statistical analysis was performed using Statistica Statsoft, version 12 package, with the Chi-squared test, ANOVA with post-hoc tests and Student’s *t* test, as appropriate. A *p* value < 0.05 was considered statistically significant.

## Results

The average age of women with OAB who completed the study (*n* = 208) was 61.2 ± 13.4 years and 68.2 % (*n* = 142) were postmenopausal. Before treatment, women with OAB reported: 11.2 (±1.7) micturitions/24 h, 1.75 (±0.74) UUI/24 h and 220.7 (±28.9) ml voided volume. The mean value of the body mass index (BMI) was 29.5 ± 5.0 (kg/m^2^) and the mean value for parity was 2.48 ± 1.26. Thirty-six participants (17.3 %) had undergone hysterectomy (30 abdominal and 6 vaginal) in the past. Women in our study group had pelvic organ prolapse (POP) stage 0 (*n* = 78, 37.5 %) and stage 1 (*n* = 130, 62.5 %) [12]. All patients, after three or more vaginal deliveries, had POP stage 1. However, the statistical analyses did not show the significant influence of POP stage on the PVR after treatment in our group. We performed uroflowmetry at the baseline and checked PVR to identify patients with bladder outlet obstruction and/or hypoactive bladder. The mean value of the maximum flow in our study was 26.2 ml/s (±8.9). We observed a moderate but statistically significant negative correlation between age and maximum flow (*r* = −0.39; *p* < 0.001).

Common comorbidities, such as hypertension, diabetes and asthma were observed in 58.6, 15.3 and 7.7 % of participants respectively. PVR volumes are summarised in Table 2. Patients were instructed how to perform CISC before the procedure; however, all women with urinary retention were re-instructed, if needed, before starting CISC. In the CISC group 2 patients demonstrated clinically significant voiding dysfunction (5 and 10 days after Botox injection) and they were unable to void spontaneously; PVRs in these patients were 250 and 290 ml respectively. Moreover, 11 patients in the CISC group demonstrated PVR greater than 350 ml at week 2. Ten of them reported noticeable voiding symptoms (splitting, hesitancy, terminal dribbling) during spontaneous micturition and/or post-micturition feeling of incomplete bladder emptying, and 1 patient demonstrated PVR > 500 ml without significant voiding symptoms.

**Table 2:** Residual volumes after Botox injections

Residual volumes in OAB patients (*n* = 208)	Follow-up (week 2), *n* (%)	Follow-up (week 4), *n* (%)	Follow-up (week 12), *n* (%)
Residual urine volume < 50 ml	106 (50.9)	115 (55.2)	188 (90.3)
Residual urine volume 51−100 ml	41 (19.7)	52 (25)	15 (7.2)
Residual urine volume 101–200 ml	35 (16.8)	28 (13.4)	4 (1.9)
Residual urine volume 201−350 ml	13 (6.2)	4 (1.9)	1 (0.4)
Urine retention requiring CISC	13 (6.2)	9 (4.3)	0

*CISC*- clean intermittent self-catheterisation

Women who required CISC and those with PVR greater than 200 ml were older in comparison with patients with PVR between 50 and 200 ml. Interestingly, patients who required CISC were also characterised by higher parity and a higher number of vaginal deliveries (Table 3). Multivariate logistic regression analysis demonstrated that three or more vaginal deliveries noticeably increased the risk of CISC (odds ratio [OR] 6.86, 95 % confidence interval [CI] 1.76–26.9, *p* < 0.01). Other factors, such as body mass index or comorbidities did not significantly influence PVR volumes and the risk of CISC. None of the patients required CICS at week 12 (Table 2). The minimum duration of CISC in our study group was 20 days and a maximum of 83 days with a mean of 45.5 days. We did not find a statistically significant association between any potential risk factors and the duration of CISC.

**Table 3:** The influence of patients’ age and route of delivery on the residual volumes at week 2 after Botox injections

Residual volumes (ml)	Age (years), mean ± SD	Parity (number), mean ± SD	Vaginal deliveries (number), mean ± SD	Caesarean sections (number), mean ± SD
Group 1 <50 ml (*n* = 106)	61.1 ± 14.5	2.5 ± 1.2	2.1 ± 1.1	0.1 ± 0.5
Group 2 51–100 ml (*n* = 41)	57.0 ± 12.1	2.3 ± 1.2	2.1 ± 1.2	0.1 ± 0.5
Group 3 101–200 ml (*n* = 35)	60.7 ± 11.8	2.3 ± 1.3	2.0 ± 1.3	0.1 ± 0.4
Group 4 201–350 ml (*n* = 13)	68.5 ± 10.2^a^	2.4 ± 1.8	2.2 ± 1.8	0.1 ± 0.3
Group 5 urine retention requiring CISC (*n* = 13)	68.8 ± 11.2^b^	3.4 ± 1.3^c^	3.2 ± 1.0^c^	0.0

^a^Group 4 vs group 2 (*p* < 0.01) and group 4 vs group 3 (*p* < 0.05)

^b^Group 5 vs group 2 (*p* < 0.01) and group 5 vs group 3 (*p* < 0.05)

^c^Group 5 vs other groups (*p* < 0.01)

## Discussion

A recent online survey asked participants with OAB about their preferences for more invasive treatment options. Participants were informed about all the potential benefits and risks of sacral neuromodulation (SNM), Botox injections and percutaneous tibial nerve stimulation (PTNS). Among 127 patients who had only experience with oral medications (anticholinergics or mirabegron) the most preferred option was PTNS (56.7 %), followed by SNM (34 %) and Botox injections (9.4 %) [13]. However, in another similar study, 74 % of women with OAB chose Botox treatment and 26 % chose SNM after failed anticholinergic treatment. Over 46 % of patients in the SNM group chose SNM to avoid the risk of urinary retention after Botox injections [14].

Patients with pelvic organ prolapse (POP) stage greater than 1 were excluded from the study. The increased prolapse stage may be a physical cause of the voiding dysfunction. It has already been published that the finding of an elevated PVR was significantly more common in patients with POP stage 2 or greater than in patients with stage 2 or less. The presence of an elevated PVR was significantly associated with symptoms of voiding difficulty and symptoms of pelvic organ prolapse. Moreover, increasing patient age was found to be a predictor of an elevated PVR only through its association with the presence of more advanced stages of POP (at least POP stage 2). The finding of an elevated PVR was found to be significantly more common in patients with POP stage 2 or greater than in patients with POP stage 1 or less [15]. On multivariate analysis the following independent predictors of raised PVR were identified: age > 55 years (OR 3.71), previous incontinence surgery (OR 4.32), a history of multiple sclerosis (OR 15.32) and pelvic organ prolapse grade 2 or greater (OR 3.61) [16]. Based on these results we did not include patients with POP stage greater than 1 to avoid the potential influence of pelvic organ prolapse on the post-treatment PVR.

Urinary retention is one of the most severe adverse events observed in OAB patients after Botox injections. Criteria for significant retention remain contentious. It has been proposed that PVR < 50 ml represents optimal emptying; therefore, we used this criterion as a basis in our post-treatment observations [17]. Moreover, a PVR > 200 ml clearly represents inadequate emptying [17, 18]. A previous study revealed that in 95 % of women aged <65 with a degree of pelvic organ prolapse ≤1 the PVR was less than 100 ml and this can be considered normal [19]. In our study we decided to compare the potential risk factors in patients with post-treatment values of PVR greater than 100 ml (101–200 ml, 201–350 ml and required CISC) with values below 50 and 100 ml. Over 6 % of patients in our study required CISC owing to urinary retention after the procedure. The rate of CISC after treatment with Botox (100 U) ranged in published studies from 2 to 31.8 % and it was associated with the patient population and the cycle of treatment [7, 11]. A meta-analysis of efficacy and adverse events after Botox trigonal vs extratrigonal injections revealed that trigonal injections were more often associated with acute urinary retention; however, this correlation was non-significant. Moreover, trigonal injections led to non-significantly higher values of PVR [20]. In a recently published series patients with OAB (wet or dry) were treated with 200 U of Botox and were advised to commence CISC in all cases with PVR > 150 ml. This strict recommendation may have been associated with the higher rates of CISC observed in this study: 23 % of all patients performed CISC. Moreover, high rates of CISC could also be associated with the 200-U dose of Botox [21]. In our study group all patients with increased PVRs who required CISC were observed during the follow-up visit at week 2. However, we had previously published that new onset urine retention could develop as late as 2 weeks after the Botox injections [22]. Therefore, patients should be informed about the potential risk of postoperative urinary retention, which can be observed even after an observation period of longer than 14 days. The mean duration of CISC in our study was 45 days and we were unable to determine factors that could significantly influence this period. Nitti et al. reported that after the first treatment cycle the median duration of CISC was 8.3 weeks and it decreased to 4.8 and then 3.1 weeks after the second and third Botox re-injections respectively [7]. Interestingly, in a previous RCT the longest reported period of CISC after treatment with Botox (100 U) was 441 days [10].

We did not observe any influence of coexisting comorbidities on the clinically important PVR after treatment, or on the risk of CISC. In another age-matched controlled study it was noted that patients with diabetes had a significantly increased (*p* = 0.007) incidence of PVR > 150 ml (60.4 %) vs non-diabetic patients (33.3 %) [23].

We found an increased risk of PVR (>200 ml) or CISC at week 2 of follow-up in patients older than 68 years. In a previous study it has been demonstrated that patients older than 61 years had a higher incidence (35.6 %) of PVR > 200 ml at week 4 after treatment. Increased PVR after treatment was also observed in patients older than 76 years (29.0 %) [24]. This observation is consistent with previous reports, which showed that elderly patients are more vulnerable to complications and adverse events. Liao and Kuo investigated the efficacy and safety of intravesical Botox treatment (100 U) in elderly (frail or not frail) in comparison with patients under the age of 65 years. Higher PVR urine volumes (defined as greater than 150 ml) after a procedure were significantly more often seen in the frail elderly group than in the other groups (60.7 % vs 39.7 and 35.7 % respectively, *p* = 0.018). Moreover, urinary retention was observed in 7 frail elderly patients (11.5 %) and in 4 (6.3 %) elderly participants who were not frail [25].

A high number of vaginal deliveries (VDs) was a significant risk factor for CISC in our study group. We did not find any previous studies that had investigated the influence of VD on complications after Botox treatment. However, it has been reported that vaginal childbirth is probably the most important factor in the aetiology of pelvic floor dysfunction, either anatomical or functional [26]. Therefore, it can be speculated that a high number of VDs could significantly affect bladder function and could potentially have an influence on the effect of pharmacological treatment.

The strengths of the study include the prospective design, the homogeneous sample, and the highly pragmatic setting. Moreover, the changes in CISC and duration of CISC were reported between follow-up visits. The resulting findings should have an immediate clinical impact in identifying patients with a potentially increased risk of urine retention after intravesical Botox injection, enabling targeted third-line treatment for women with OAB. Limitations of this study include the lack of a placebo control group and the lack of generalisability to male patients. In our opinion, the first 2 weeks after Botox injections are crucial for the observation of PVR and voiding symptoms. Therefore, patients should be informed that they could expect these adverse events relatively early in the post-injection period. The risk of new onset urine retention in our study has completely disappeared 2 weeks after Botox injections. Based on our results of the way in which the PVRs have changed in time, we can conclude that OAB patients should be optimally assessed during the first 2 weeks after Botox injections.

Elderly women and multiparous women are at an increased risk of urinary retention after 100-U intravesical Botox injections. However, all patients, independent of age or parity, should be warned before receiving Botox injections about the potential risk of urine retention after treatment. The decision to instigate CISC should be made based on patients’ subjective significant voiding dysfunction symptoms coexisting with increased PVR (<350 ml) or on the significant increase in PVR measurements (>350 ml).
